# ZIKV-envelope proteins induce specific humoral and cellular immunity in distinct mice strains

**DOI:** 10.1038/s41598-022-20183-x

**Published:** 2022-09-21

**Authors:** Victória Alves Santos Lunardelli, Juliana de Souza Apostolico, Higo Fernando Santos Souza, Fernanda Caroline Coirada, Jéssica Amaral Martinho, Renato Mancini Astray, Silvia Beatriz Boscardin, Daniela Santoro Rosa

**Affiliations:** 1 Department of Microbiology, Immunology and Parasitology, Federal University of São Paulo (UNIFESP/EPM), São Paulo, Brazil; 2 Department of Parasitology, Institute of Biomedical Sciences, University of São Paulo, São Paulo, Brazil; 3 Viral Immunology Laboratory, Butantan Institute, São Paulo, Brazil; 4 Institute for Investigation in Immunology (III), INCT, São Paulo, Brazil

**Keywords:** Protein vaccines, Adjuvants

## Abstract

Recent outbreaks of Zika virus (ZIKV) infection have highlighted the need for a better understanding of ZIKV-specific immune responses. The ZIKV envelope glycoprotein (E_ZIKV_) is the most abundant protein on the virus surface and it is the main target of the protective immune response. E_ZIKV_ protein contains the central domain (EDI), a dimerization domain containing the fusion peptide (EDII), and a domain that binds to the cell surface receptor (EDIII). In this study, we performed a systematic comparison of the specific immune response induced by different E_ZIKV_ recombinant proteins (E_ZIKV_, EDI/II_ZIKV_ or EDIII_ZIKV_) in two mice strains. Immunization induced high titers of E-specific antibodies which recognized ZIKV-infected cells and neutralized the virus. Furthermore, immunization with E_ZIKV_, EDI/II_ZIKV_ and EDIII_ZIKV_ proteins induced specific IFNγ-producing cells and polyfunctional CD4^+^ and CD8^+^ T cells. Finally, we identified 4 peptides present in the envelope protein (E_1–20_, E_51–70_, E_351–370_ and E_361–380_), capable of inducing a cellular immune response to the H-2K^d^ and H-2K^b^ haplotypes. In summary, our work provides a detailed assessment of the immune responses induced after immunization with different regions of the ZIKV envelope protein.

## Introduction

Research on the immune response to the Zika virus (ZIKV), a mosquito-borne flavivirus, has increased after recent outbreaks^1–3^. Unlike other flaviviruses, ZIKV transmission was also observed through non-vector transmission (sexual, transfusional, and vertical)^4,5^. ZIKV infection in pregnant women has been associated with congenital malformations (brain calcification, microcephaly, and spontaneous abortion), characterizing the congenital Zika syndrome (CZS)^6–9^, while in adults it is associated with Guillain-Barré syndrome (GBS)^10,11^. The rapid global spread of ZIKV and the suspected association with serious neurological implications have led to the urgent need for an effective vaccine and specific treatment against the virus. Even with scientific efforts, little is known about the ZIKV-specific immune response and there are still no licensed therapeutic or prophylactic vaccines against ZIKV.

ZIKV has an 11 kb positive-sense single-stranded RNA (ssRNA) genome that encodes a single polyprotein that is cleaved into three structural proteins (Capsid (C), Premembrane/Membrane (prM/M) and Envelope (E)) and seven non-structural proteins (NS1, NS2A, NS2B, NS3, NS4A, NS4B, and NS5) involved in virus replication and assembly^12,13^. The envelope protein mediates viral assembly, binding to cell receptors and is essential for the subsequent fusion of the membrane involved in virus entry into the target cell^14^. Similar to other flaviviruses, the ZIKV E protein contains three distinct domains: the central domain (EDI), the domain responsible for dimerization that contains the fusion peptide (EDII), and the domain that binds to the cell surface receptor (EDIII)^15^.

Regarding the humoral immune response induced by flaviviruses, several studies have shown that protein E is highly immunogenic. Furthermore, protein E is the main target of several ZIKV-specific neutralizing antibodies^16,17^ that also confer protection in animal models of infection^18,19^. Several E-specific monoclonal antibodies (mAbs) inhibited ZIKV infection^19–22^. In addition, passive transfer of E-specific mAb reduced vertical transmission and mortality in mice^17^. Although antibodies are the main correlates of protection against ZIKV infection, T cell immunity also plays an important role in controlling virus replication^23^. It has already been demonstrated that the absence of CD8^+^ T cells during ZIKV infection is capable of increasing mortality in mice^24^. CD4^+^ T cells also participate in the generation of protective immunity, since their depletion reduced the induction of anti-ZIKV antibodies^25,26^ and CD8^+^ T cell responses^27^.

In this study, we investigated the induction of humoral and cellular immune responses after immunization of BALB/c and C57Bl/6 mice with the ZIKV-envelope protein (E_ZIKV_) and its domains (EDI/II_ZIKV_ and EDIII_ZIKV_). We observed that immunization with EDI/II_ZIKV_ and EDIII_ZIKV_ proteins induced high titers of specific antibodies, which recognized ZIKV-infected cells and neutralized the virus. In addition, immunization with the proteins was able to induce specific IFNγ-producing cells and polyfunctional CD4^+^ and CD8^+^ T cell responses. We also mapped immunodominant epitopes in ZIKV-envelope region, and identified four peptides (E_1–20_, E_51–70_, E_351–370_ and E_361–380_) capable of inducing specific T cells to the H-2K^d^ and H-2K^b^ haplotypes.

## Results

### Production of recombinant envelope proteins

After alignment of the 69 ZIKV isolates we observed 96.75% of homology among the amino acid sequences. Additionally, across the different isolates, it was possible to identify 13 different sequences with at least one amino acid mutation corresponding to 18.9% of the ZIKV isolates. The artificial gene corresponding to the consensus sequence of the ZIKV envelope was synthetized (Supplementary Table 1) and cloned to produce the entire ectodomain of the envelope recombinant protein (E_ZIKV_) (amino acids 291–690) and its domains EDI/II_ZIKV_ (aa 291–600) and EDIII_ZIKV_ (aa 601–690) (Supplementary Fig. 1a). The recombinant E_ZIKV_, EDI/II_ZIKV_ and EDIII_ZIKV_ were purified by affinity chromatography (Supplementary Fig. 1b) and further recognized by anti-His tag (Supplementary Fig. 1c). We also performed a Dot Blot with the expressed proteins using an anti-flavivirus 4G2 monoclonal antibody, which recognizes a conformational epitope present in the E protein domain II fusion loop. The 4G2 specifically recognized E_ZIKV_ and EDI/II_ZIKV_ proteins, but not the EDIII_ZIKV_ domain, suggesting that the expressed proteins retained the correct conformation (Supplementary Fig. 1d).

### Immunization with ZIKV envelope proteins induces a potent specific humoral immune response in BALB/c and C57Bl/6 mice

**Figure 1:**
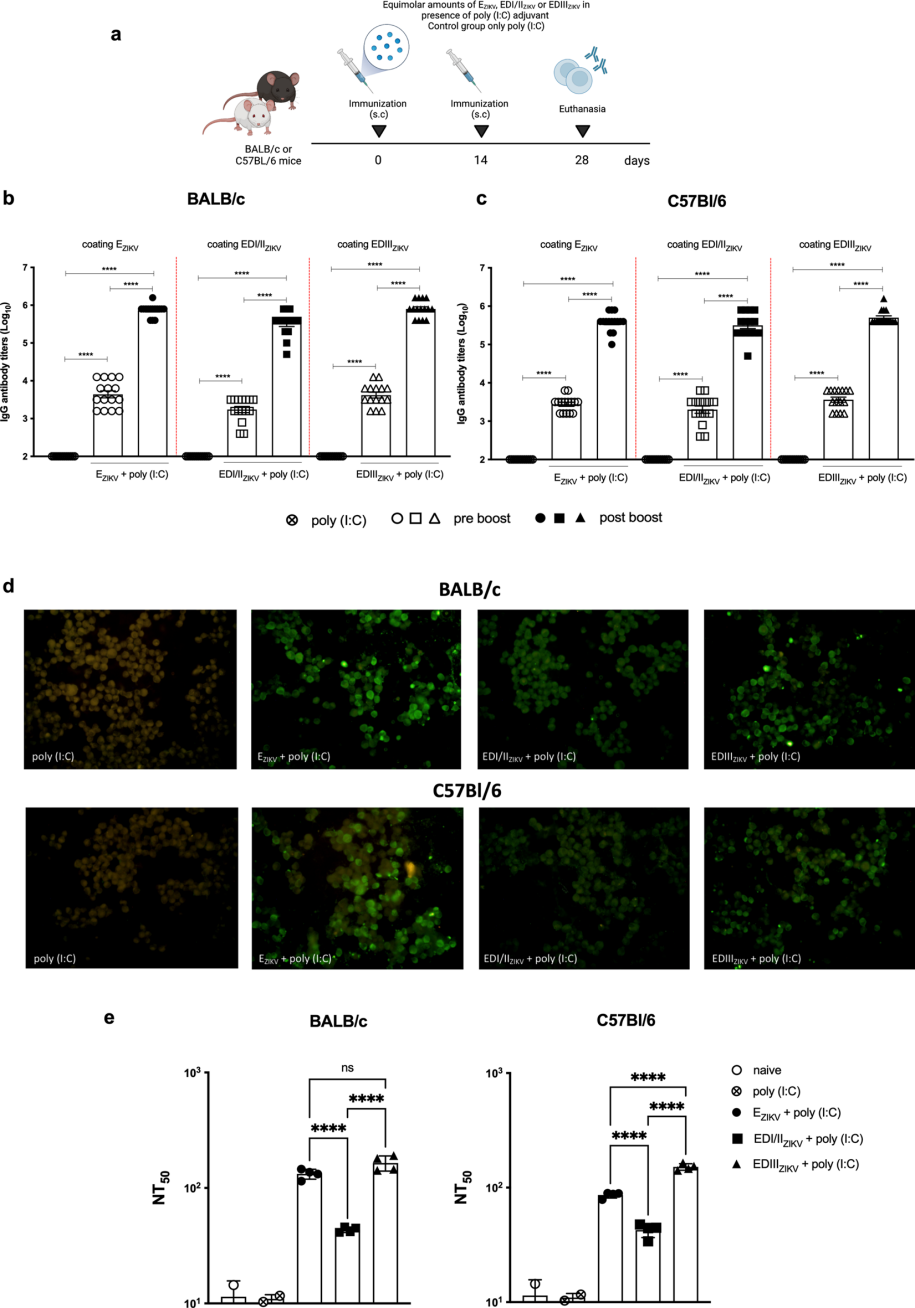
Specific humoral immune response elicited after immunization with E_ZIKV_, EDI/II_ZIKV_ or EDIII_ZIKV_ recombinant protein in two different mouse strains. (**a**) Immunization Strategy (created with BioRender.com). BALB/c or C57Bl/6 mice (n = 3 control groups and n = 4 experimental groups) were immunized subcutaneously twice with equimolar amounts of the E_ZIKV_, EDI/II_ZIKV_ or EDIII_ZIKV_ combined with 50 μg poly(I:C). Control groups received only poly(I:C). Mice were bled 14 days after each dose to evaluate humoral response. (**b**, **c**) Total specific-IgG antibody titers on a logarithm scale (Log_10_) in the sera of (**b**) BALB/c or (**c**) C57Bl/6 mice. Empty symbols represent pre boost serum and filled symbols represent post boost serum. Post boost sera was inactivated in order to assess their ability to (**d**) recognize ZIKV-infected Vero cells (MOI = 0.1) or (**e**) neutralize ZIKV. (**d**) For IFA assay, mouse serum and goat anti-mouse IgG conjugated with fluorescein isothiocyanate was used as primary and secondary antibodies respectively. (**e**) For PRNT, sera were incubated with 100 PFU of ZIKV and the neutralization capacity were represented by 50% of viral neutralization (NT50). Data represent mean ± SEM of 4 independent experiments. Statistical significance was measured by One-way ANOVA followed by Tukey’s post hoc test, *p < 0.05, **p < 0.01, ***p < 0.001, ****p < 0.0001.

Next, we assessed the immunogenicity of the subunit vaccines in BALB/c and C57Bl/6 mice. For this purpose, mice received two doses with an equimolar amount of E_ZIKV_, EDI/II_ZIKV_ or EDIII_ZIKV_ administered subcutaneously in the presence of adjuvant poly (I:C) (Fig. 1a). Fourteen days after each dose, sera were tested for reactivity against ZIKV envelope proteins. Sera from all groups immunized with the recombinant proteins presented antigen-specific antibodies in both BALB/c (Fig. 1b) and C57Bl/6 (Fig. 1c) strains. Furthermore, after the boost, the antibody titers increased significantly. A head-to-head comparison after the second dose revealed that BALB/c mice immunized with E_ZIKV_ or EDIII_ZIKV_ presented slightly higher antibody titers when compared to animals that received EDI/II_ZIKV_ (Supplementary Fig. 2a). However, no significant differences were observed between antibody titers in C57Bl/6 mice that received E_ZIKV_, EDI/II_ZIKV_ or EDIII_ZIKV_. The humoral response against different domains was also analyzed by ELISA (Supplementary Fig. 2b). Anti-EDI/II_ZIKV_ and anti-EDIII_ZIKV_ humoral immune responses showed high specificity, while anti-E_ZIKV_ antibodies reacted with EDI/II_ZIKV_ and EDIII_ZIKV_ proteins. In addition, an immunofluorescence assay (IFA) showed that sera from mice immunized with different ZIKV envelope proteins recognized ZIKV-infected cells (Fig. 1d). In contrast, the control group immunized with only poly (I:C) did not produce specific antibodies against the ZIKV envelope proteins (Fig. 1b,c) and was unable to recognize ZIKV-infected cells (Fig. 1d).

### Immunization with different envelope proteins induces neutralizing antibodies against ZIKV infection

To evaluate the quality of the antibodies generated, we performed standard plaque reduction neutralization testing (PRNT). In BALB/c mice, we observed that the serum of all immunized groups reduced ZIKV infection (Fig. 1e). However, we can observe that the antibody titers that promoted 50% of viral neutralization (NT50) were higher in the group immunized with E_ZIKV_ or EDIII_ZIKV_ in the presence of poly (I:C). The lowest NT50 values were observed in the groups immunized with EDI/II_ZIKV_. For C57Bl/6 mice, we observed a similar profile, but immunization with EDIII_ZIKV_ led to a higher production of neutralizing antibodies when compared to immunization with E_ZIKV_. In contrast, the naïve or adjuvant group did not display significant neutralizing antibodies against the virus. So far, our data demonstrate that the EDIII component induces the most robust humoral immune response against ZIKV.

### Subunit vaccines induced IFNγ-producing cells against recombinant ZIKV envelope proteins in different mouse strains

**Figure 2:**
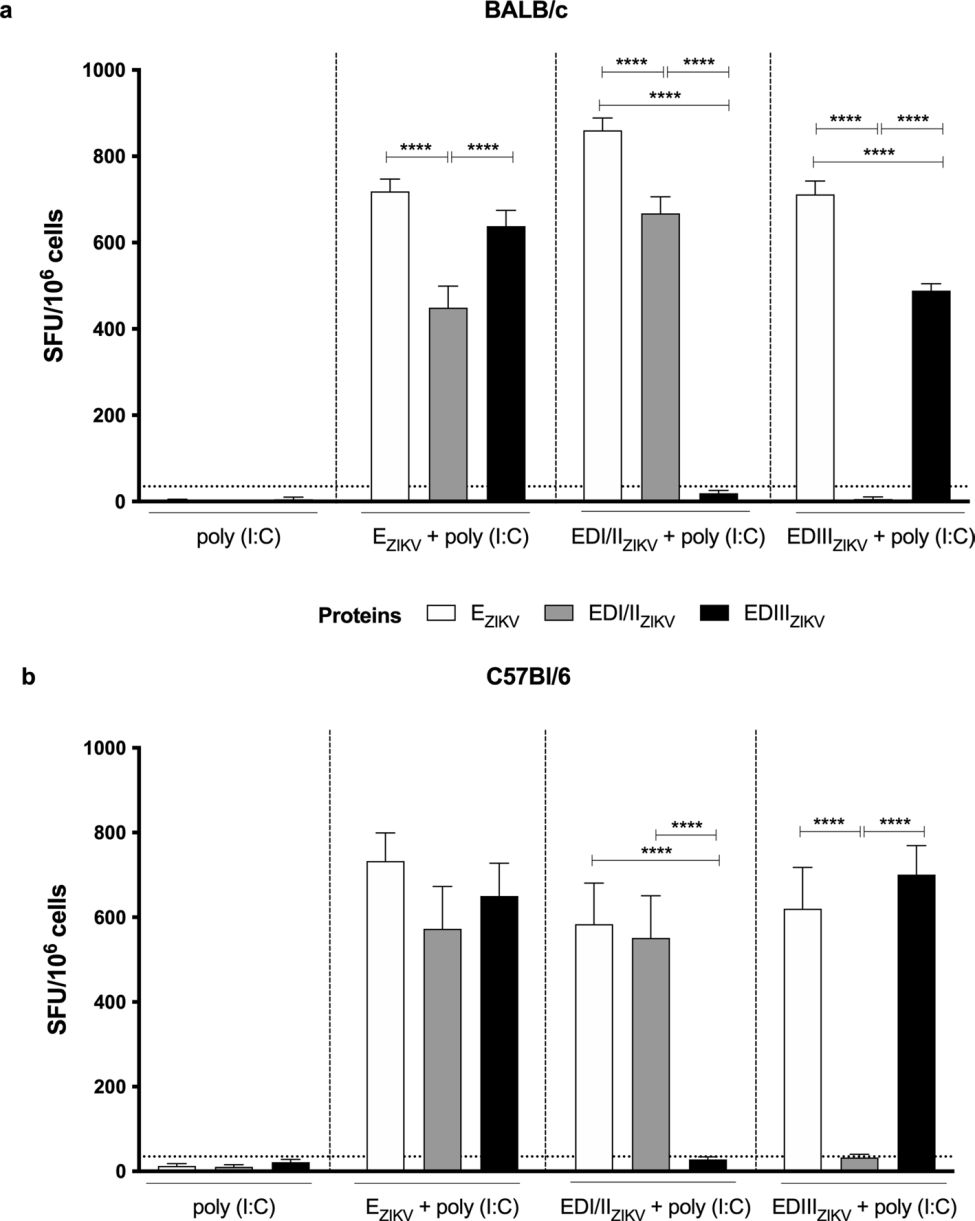
Specific IFNγ-producing cells after immunization with ZIKV-envelope proteins. Analysis of the specific cellular immune response after immunization of (**a**) BALB/c or (**b**) C57Bl/6 mice as described in Fig. 1a. Fifteen days after the boost, the splenocytes were cultured in the presence of equimolar amount of recombinant envelope proteins for 18 h to evaluate the number of IFN-γ producing cells by ELISpot assay. SFU: spot forming units. Statistical significance was measured by Two-way ANOVA followed by Tukey’s post hoc test, *p < 0.05, **p < 0.01, ***p < 0.001, ****p < 0.0001. Data represent mean ± SEM of 4 independent experiments.

Next, we evaluated whether immunization with E_ZIKV_, EDI/II_ZIKV_ and EDIII_ZIKV_ mixed with poly (I:C) in BALB/c and C57Bl/6 mice would induce cellular-mediated immunity. Splenocytes harvested fifteen days after boost were incubated with recombinant ZIKV envelope proteins to assess specific cytokine production. Figure 2 shows IFNγ-producing cells by ELISpot. In BALB/c (Fig. 2a) or C57Bl/6 (Fig. 2b) splenocytes, we observed that the group that received E_ZIKV_ + poly (I:C) presented IFNγ-producing cells when stimulated with all recombinant ZIKV proteins. On the contrary, splenocytes from mice immunized with EDI/II_ZIKV_ + poly (I:C) or EDIII_ZIKV_ + poly (I:C) only induced IFNγ-producing cells against E_ZIKV_ and EDI/II_ZIKV_ or against E_ZIKV_ and EDIII_ZIKV_, respectively. We also observed a lower number of IFNγ-producing cells in splenocytes from BALB/c mice stimulated with EDI/II_ZIKV_ when compared to the entire protein or domain III (Fig. 2a). Moreover, BALB/c mice immunized with adjuvanted EDI/II_ZIKV_ or EDIII_ZIKV_ presented higher number of IFNγ-producing cells against E_ZIKV_ when compared to the correlated domain used in immunization (Fig. 2a). Furthermore, the administration of poly (I:C) alone did not induce IFNγ-producing cells.

### T-cell epitope coverage

**Figure 3:**
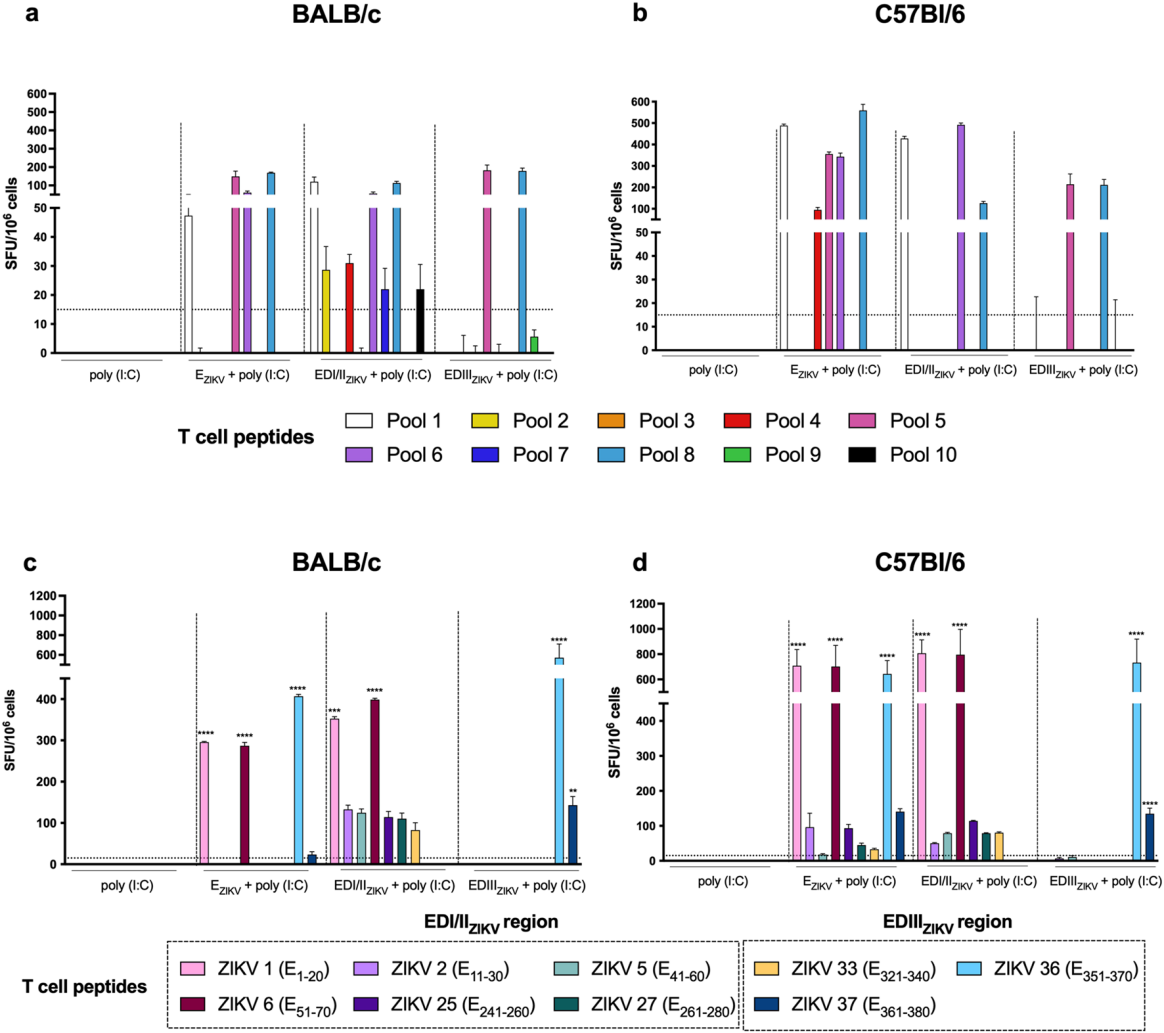
Mapping of T cell epitopes after immunization with recombinant ZIKV envelope proteins. Analysis of the specific cellular immune response after immunization of (**a**, **c**) BALB/c or (**b**, **d**) C57Bl/6 mice as described in Fig. 1a. Fifteen after the second dose, the spleen of each animal was removed and the splenocytes were cultured in the presence of 10 mg/mL of the (**a**, **b**) pool of ZIKV peptides or (**c**, **d**) individual peptides to evaluate the number of IFNγ-producing cells by ELISpot assay. SFU: spot forming units. Statistical significance was measured by Two-way ANOVA followed by Tukey’s post hoc test, *p < 0.05, **p < 0.01, ***p < 0.001, ****p < 0.0001. Data represent mean ± SEM of 3 independent experiments.

To evaluate the breadth of T cell responses and map the coverage of ZIKV-derived epitopes, a total of 39 peptides (20 amino acids overlapping 12-mer) were synthetized comprising the ZIKV E protein sequence (aa 291–690) conserved among 69 ZIKV isolates (GenBank accession numbers available at Supplementary Table 1). An optimized matrix containing 10 peptide pools was generated using *Deconvolute This!* Software (Supplementary Table 2)^28^. Splenocytes from BALB/c immunized with E_ZIKV_ + poly (I:C) presented IFNγ-producing cells to peptides presented in pools 1, 5, 6 and 8 (Fig. 3a). In addition to those covered by the BALB/c strain, C57Bl6 mice that received E_ZIKV_ + poly (I:C) also showed an IFNγ response against pool 4 (Fig. 3b). BALB/c mice immunized with EDI/II_ZIKV_ + poly (I:C) presented a higher number of IFNγ-producing cells when stimulated by pools 1, 6, and 8 (Fig. 3a) but also against pools 2, 4, 7 and 10. On the other hand, IFNγ response by the same subunit vaccine in C57Bl/6 mice was exclusively directed to peptides present in pools 1, 6, and 8 (Fig. 3b). In both mouse strains, immunization with EDIII_ZIKV_ + poly (I:C) induced an IFNγ-response against pools 5 and 8 (Fig. 3a,b).

From those results, we selected a total of 9 out of 39 potential peptides for further evaluation. ZIKV peptides from ectodomain I/II: (ZIKV 1 (E_1–20_), ZIKV 2 (E_11–30_), ZIKV 5 (E_41–60_), ZIKV 6 (E_51–70_), ZIKV 25 (E_241–260_) and ZIKV 27 (E_261–280_)) and ectodomain III: (ZIKV 33 (E_321–340_), ZIKV 36 (E_351–370_) and ZIKV 37 (E_361–380_)) were individually tested (Fig. 3c,d). Notably, immunization with E_ZIKV_ + poly (I:C) induced IFNγ-producing cells against two peptides present in domain I/II (ZIKV 1 (E_1–20_) and ZIKV 6 (E_51–70_)) and two present in domain III (ZIKV 36 (E_351–370_) and ZIKV 37 (E_361–380_)) in both BALB/c (Fig. 3c) and C57Bl/c (Fig. 3d) mice, albeit with a higher magnitude in C57Bl/6 mice. Furthermore, the same peptides induced responses after immunization with the respective domains. In mice immunized with EDI/II_ZIKV_ + poly (I:C) or EDIII_ZIKV_ + poly (I:C), the response was mainly directed against ZIKV 1 (E_1–20_) and ZIKV 6 (E_51–70_) or ZIKV 36 (E_351–370_) and ZIKV 37 (E_361–380_), respectively. The most immunogenic peptides in the ZIKV envelope amino acid sequence, ZIKV 1 (E_1–20_), ZIKV 6 (E_51–70_), ZIKV 36 (E_351–370_) and ZIKV 37 (E_361–380_) are represented in Supplementary Fig. 3a.

### Subunit vaccines induce specific T cells that proliferate and produce pro-inflammatory cytokines

**Figure 4:**
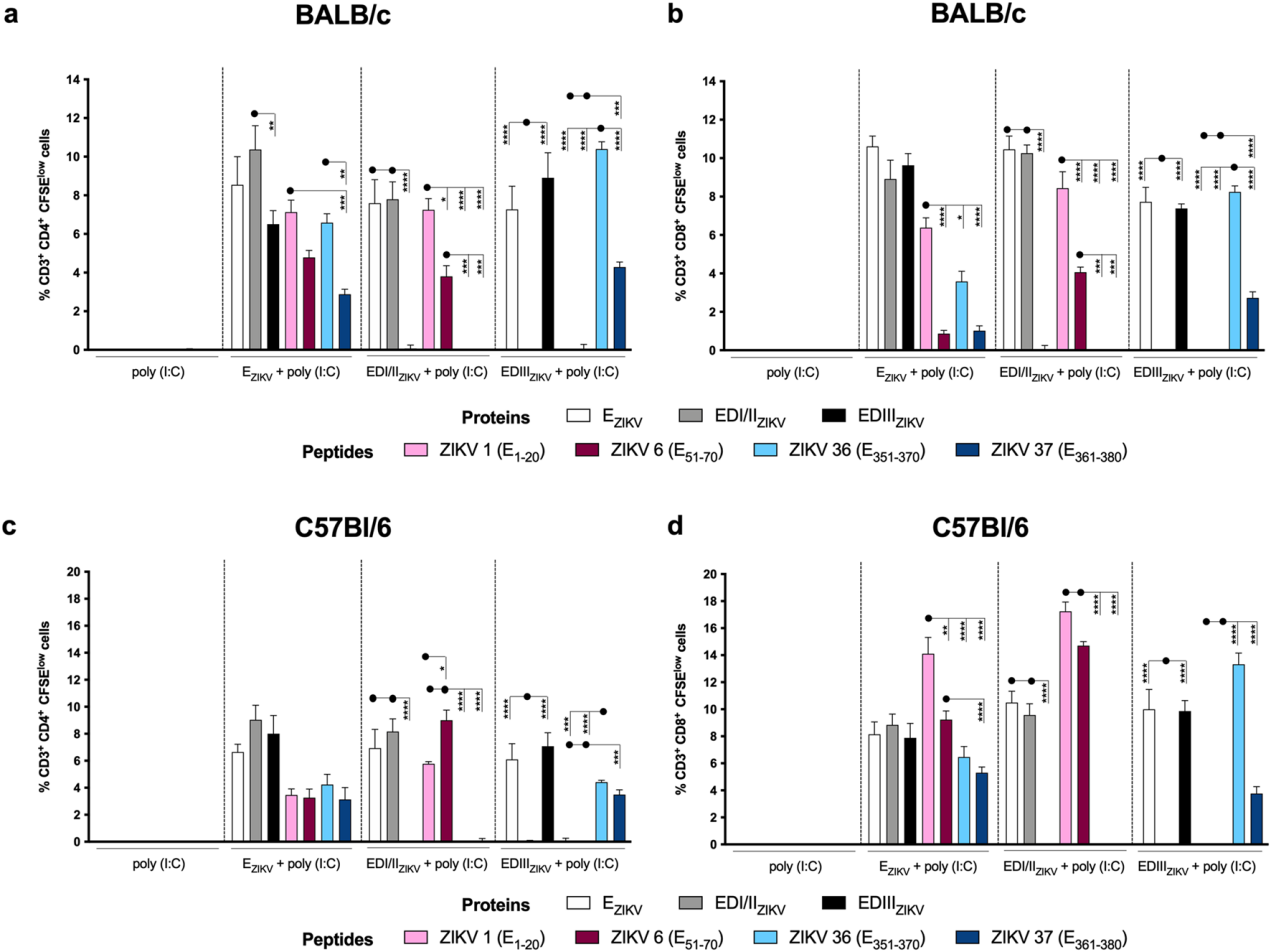
Immunization with recombinant ZIKV envelope proteins induces proliferation of specific CD4^+^ and CD8^+^ T cells. Analysis of CD4^+^ and CD8 + T cell proliferation after immunization of (**a**, **b**) BALB/c or (**b**, **d**) C57Bl/6 mice as described in Fig. 1a. Fifteen days after the second dose, the spleen of each animal was removed and the splenocytes were labeled with CFSE (1.25 μM) and cultured in the presence of equimolar amounts of recombinant proteins or 5 μg/mL of the individual peptides for 5 days. After labeling with fluorochrome-conjugated anti-CD3, -CD4 and -CD8, cells were analyzed by flow cytometry (representative gating strategies shown in Supplementary Fig. 3b). Initially, a gate was performed on CD3^+^ cells (T lymphocytes), followed by gates on CD4^+^ and CD8^+^ populations. Within the two T cells subpopulations (**a**, **c**) CD3^+^CD4^+^ and (**b**, **d**) CD3^+^CD8^+^, the decrease in CFSE fluorescence intensity was evaluated. The frequency of cell proliferation was calculated by subtracting the values from the culture of unstimulated cells. Statistical significance was measured by Two-way ANOVA followed by Tukey’s post hoc test, *p < 0.05, **p < 0.01, ***p < 0.001, ****p < 0.0001. Data represent mean ± SEM of 2 independent experiments.

**Figure 5:**
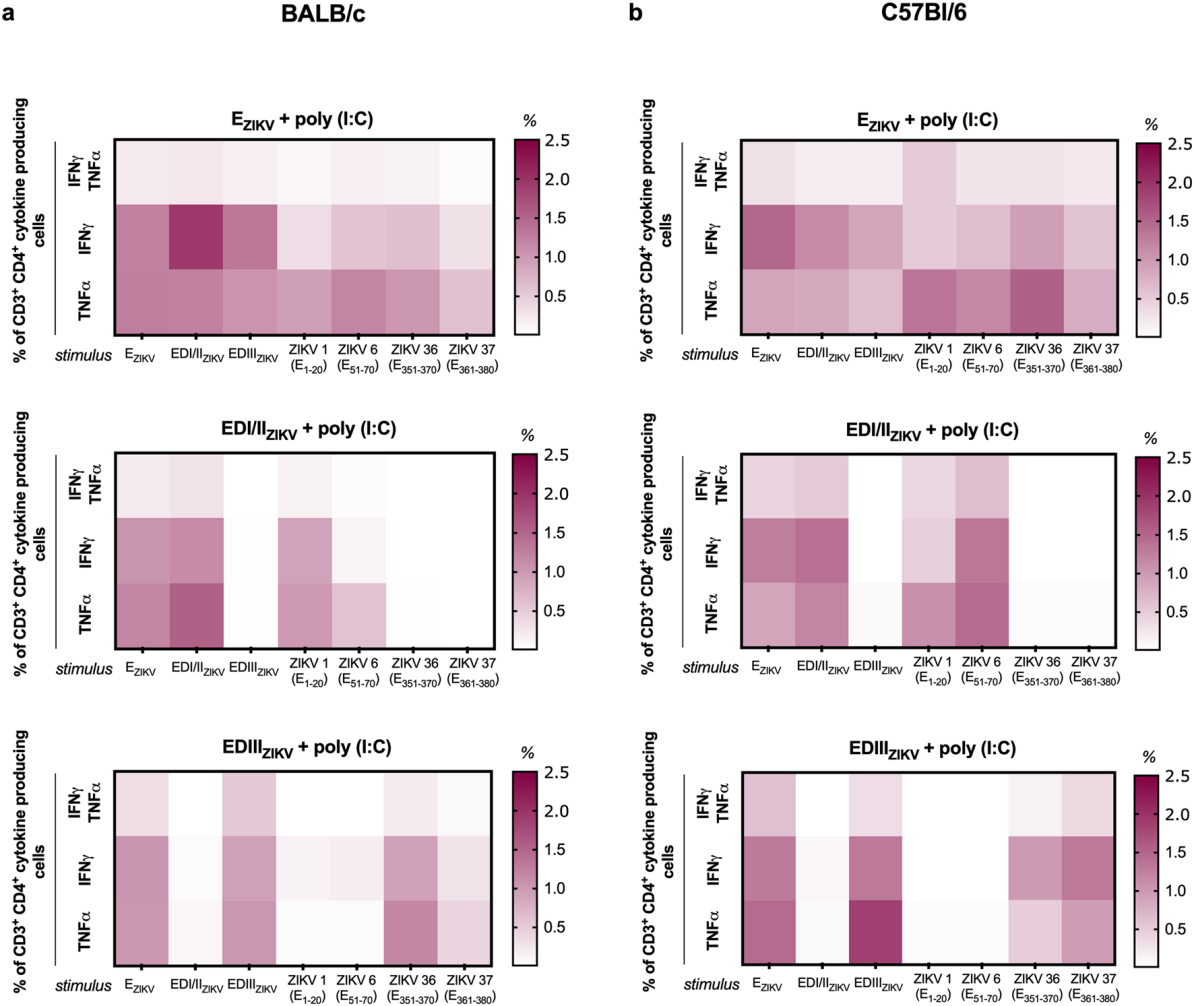
Immunization with recombinant ZIKV envelope proteins induces polyfunctional CD4^+^ T cells. Analysis of polyfunctional cells after immunization of (**a**) BALB/c or (**b**) C57Bl/6 mice as described in Fig. 1a. Fifteen days after the second dose, the spleen of each animal was removed and cultured in the presence of equimolar amounts of recombinant proteins or 5 μg/mL of the individual peptides. For the detection of cytokine-producing T cells, the cells were restimulated on the 4th day for 12 h in the presence of recombinant proteins, anti-CD28 and brefeldin A. Cells were stained with anti-CD3 and -CD4, then permeabilized and labeled for intracellular cytokines. After selecting the T cell populations that produce cytokines (representative gating strategies are shown in Supplementary Fig. 3b), a Boolean combination was created using the FlowJo software to determine the frequency of each response based on all possible combinations of CD4^+^ T cell cytokine producers. Heatmap was used to determine the frequency of CD4^+^ T cells that produce IFNγ and TNFα when stimulated with the different proteins (columns). The frequency of CD4 + T cells that produce cytokines was calculated by subtracting the values from the unstimulated cell culture.

**Figure 6:**
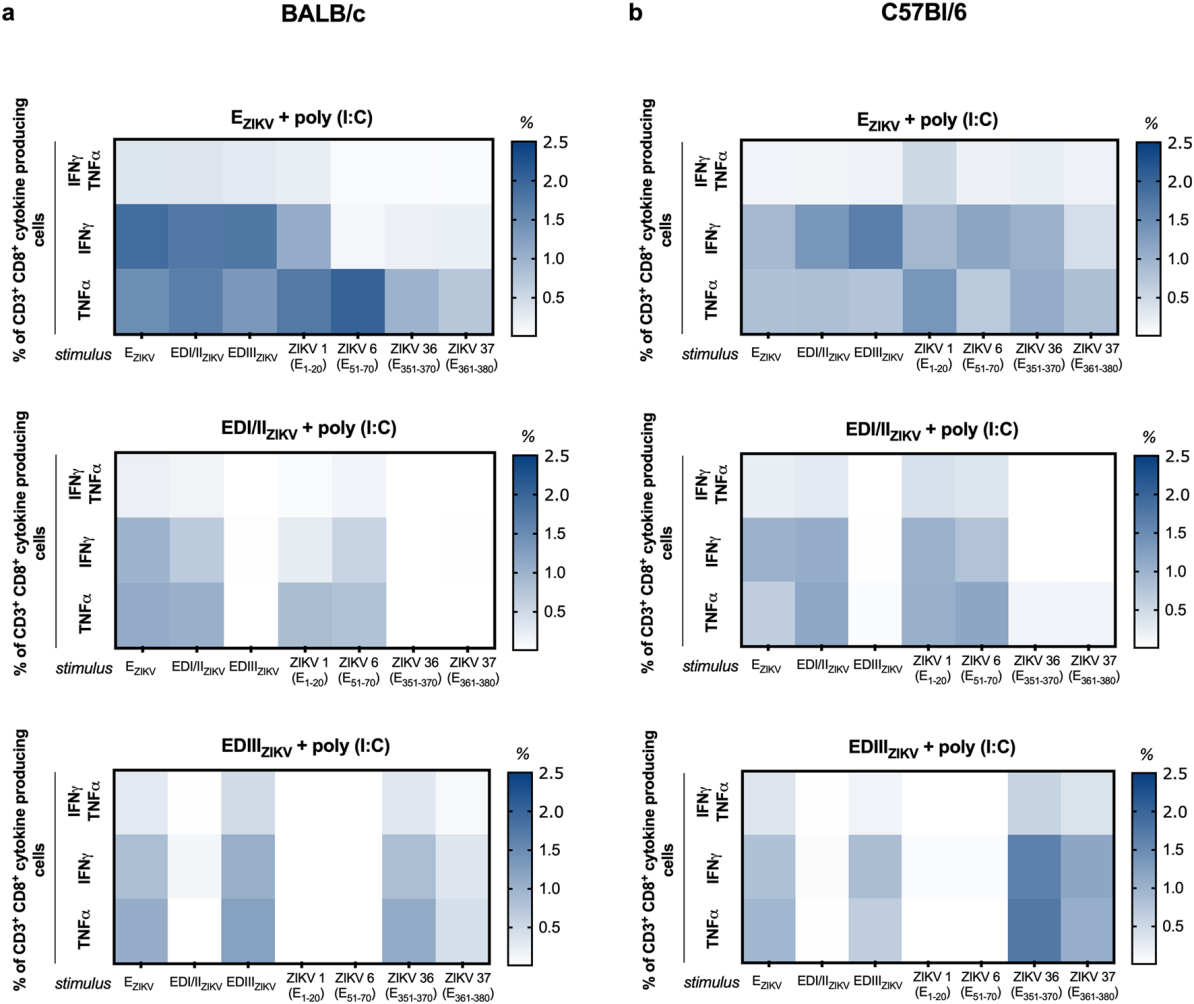
Immunization with recombinant ZIKV envelope proteins induces polyfunctional CD8^+^ T cells. Analysis of polyfunctional cells after immunization of (**a**) BALB/c or (**b**) C57Bl/6 mice as described in Fig. 1a. Fifteen days after the second dose, the spleen of each animal was removed and cultured in the presence of equimolar amounts of recombinant proteins or 5 μg/mL of the individual peptides. For the detection of cytokine-producing T cells, the cells were restimulated on the 4th day for 12 h in the presence of recombinant proteins, anti-CD28 and brefeldin A. Cells were stained with anti-CD3 and -CD8, then permeabilized and labeled for intracellular cytokines. After determining the populations of T cells that produce cytokines (representative gating strategies are shown in Supplementary Fig. 3b, a Boolean combination was created using the FlowJo software to determine the frequency of each response based on all possible combinations of CD8^+^ T cells cytokine producers. Heatmap was used to determine the frequency of CD8^+^ T cells that produce IFNγ and TNFα when stimulated with the different proteins (columns). The frequency of cells that produce cytokines was calculated by subtracting the values from the unstimulated cell culture.

Next, we sought to evaluate whether immunization with different the subunit vaccines induce Env-specific CD4^+^ and CD8^+^ T cells able to proliferate (Fig. 4) or produce IFNγ and/or TNFα (Figs. 5 and 6) (representative gating strategies in Supplementary Fig. 3b). In BALB/c mice, immunization with E_ZIKV_ induced proliferation of CD4^+^ (Fig. 4a) and CD8^+^ (Fig. 4b) T cells against the proteins E_ZIKV_, EDI/II_ZIKV_ and EDIII_ZIKV_, as well as against the peptides present in domain I/II (ZIKV 1 (E_1–20_) and ZIKV 6 (E_51–70_)) and domain III (ZIKV 36 (E_351–370_) and ZIKV 37 (E_361–380_)). The proliferation of CD4^+^ and CD8^+^ T cells in mice immunized with the domains (EDI/II_ZIKV_ and EDIII_ZIKV_) was specific, i.e., targeted to the E_ZIKV_ protein and to the domain used in immunization. The same phenomenon was observed for CD4^+^ (Fig. 4c) and CD8^+^ (Fig. 4d) T cell proliferation in C57BL/6 mice. It is worth mentioning that the frequency of proliferating CD8^+^ T cells was higher in C57Bl/6 (Fig. 4d) when compared to the BALB/c strain (Fig. 4b).

Subsequently, we analyzed the cytokine profile of specific T lymphocytes. In both BALB/c (Figs. 5a, 6a and Fig. S4a) and C57Bl/6 (Figs. 5b, 6b and Fig. S4b) mice, immunization with E_ZIKV_ induced CD4^+^ and CD8^+^ T cells able to produce IFNγ and TNFα alone or simultaneously against all the stimuli (E_ZIKV_, EDI/II_ZIKV_, EDIII_ZIKV_ recombinant proteins; peptides ZIKV 1 (E_1–20_), ZIKV 6 (E_51–70_), ZIKV 36 (E_351–370_) and ZIKV 37 (E_361–380_)). As expected, immunization with the EDI/II_ZIKV_ and EDIII_ZIKV_ domains was able to induce a polyfunctional response only against the recombinant protein E_ZIKV_ or the specific domains used in the immunization. In contrast, the group immunized with the adjuvant alone did not induce a significant frequency of CD4^+^ and CD8^+^ T cells that proliferated or produced cytokines. These data suggest that immunization with ZIKV envelope proteins was able to induce specific T cell responses. Furthermore, this result suggests the ability of ZIKV 1 (E_1–20_), ZIKV 6 (E_51–70_), ZIKV 36 (E_351–370_) and ZIKV 37 (E_361–380_) peptides to bind to H-2K^d^ and H-2K^b^ haplotypes and induce T cell immunity.

## Discussion

Recent outbreaks of ZIKV and the possible sequelae due to neurological morbidity in newborns and adults^1,3^ led to substantial changes in public health policies. There is no vaccine or treatment against the virus, highlighting the need for a better understanding of specific immunity. The ZIKV envelope protein is essential for virus entry into cells and is the main antigen that triggers host immune responses^14^. Here, we evaluated the ability of the recombinant protein E_ZIKV_ and its domains (EDI/II_ZIKV_ and EDIII_ZIKV_) to induce humoral and cellular immune responses in two different mouse strains. C57Bl/6 and BALB/c mice were immunized with equimolar amounts of the recombinant proteins in the presence of the adjuvant poly (I:C).

The development of antibodies is considered fundamental against viral infections^29^. In flaviviruses, E protein, prM, and NS1 are the main targets of the antibody response^30^. We observed that the proteins E_ZIKV_, EDI/II_ZIKV_ and EDIII_ZIKV_ were highly immunogenic, inducing specific antibodies that neutralized the virus. However, antibodies induced after boost with E_ZIKV_ or EDIII_ZIKV_ displayed greater neutralizing capacity when compared to anti-EDI/II_ZIKV_ antibodies. Previous studies demonstrate that domain III of the envelope protein is the main target for neutralizing antibodies ^20,21,31–36^. In addition, domain III does not induce antibody-dependent enhancement (ADE) and protected mice after challenge with ZIKV^33,35^. Furthermore, we observed that antibodies generated against immunization with the recombinant protein E_ZIKV_ were able to recognize the EDI/II_ZIKV_ and EDIII_ZIKV_ domains, with higher titers against domain I/II when compared to domain III. Sera from animals immunized with EDI/II_ZIKV_ or EDIII_ZIKV_ specifically recognized the same administered protein as well as the entire E_ZIKV_ protein but were unable to recognize the distinct domain. Within the E-specific response, the relative proportion of DI/II versus DIII antibodies showed considerable variability in serological studies with West Nile and Dengue (DENV) viruses, with an overall prevalence of antibodies against domains I/II^34,37–40^.

During ZIKV infection, although there is a prevalence of antibodies against domains I/II that reach a peak during the beginning of the infection and fall over time, antibodies generated against domain III are more neutralizing and persist for a long period of time^41^. Here, sera from EDIII_ZIKV_-immunized mice presented greater ability to neutralize ZIKV than those induced after immunization with E_ZIKV_ or EDI/II_ZIKV_ in the C57Bl/6 strain. However, for BALB/c mice, sera from E_ZIKV_ or EDIII_ZIKV_ mice showed the same neutralization capacity. In fact, protective anti-ZIKV antibody titers have already been observed in BALB/c mice after immunization with a truncated E_ZIKV_ protein ^42^. Likewise, C57BL/6 mice immunized with EDIII_ZIKV_ showed protective humoral immunity^35^. Yang et al. demonstrated that immunization with virus-like particles (VLP) containing EDIII_ZIKV_ in the presence of poly (I:C) induced a strong humoral response in C57BL/6 mice^36^.

Several studies demonstrated the important role of the cellular immune response against flaviviruses. The absence of CD8^+^ T cells during ZIKV infection increases mortality in mice^24^. DENV-specific CD8^+^ T cells induce cross-protection against ZIKV infection, including during pregnancy^27^. CD8^+^ T cells were shown to be essential to control yellow fever virus (YFV) and ZIKV infection in mice deficient in B lymphocytes^43,44^. CD4^+^ T cells also participate in the generation of protective immunity, as their depletion reduced the generation of anti-ZIKV antibodies^25,26^ and CD8^+^ T cell responses^27^. Recently, CD4^+^ T cells and IFNγ signaling have been shown to play a central role in protection during Zika virus infection^45^. Transfer experiments revealed that CD4^+^ T cells are required to protect against lethal challenge by ZIKV^46^. Furthermore, in a murine model of neuroinvasive ZIKV infection, the absence of CD4^+^ T cells leads to more neurological sequelae and increased viral titers in the central nervous system^46^. Indeed, the presence of polyfunctional CD4^+^ T cell responses is also implicated in protection against Japanese encephalitis virus (JEV) infection^47^, and is a hallmark after effective YFV vaccination^48,49^. We observed that immunization with E_ZIKV_, EDI/II_ZIKV_ and EDIII_ZIKV_ proteins induced specific IFNγ-producing cells and polyfunctional CD4^+^ and CD8^+^ T cell responses.

Several studies have been carried out to identify immunodominant epitopes recognized by CD4^+^ and CD8^+^ T cells during flavivirus infections, particularly in DENV infection^50^. In DENV-infected patients and participants vaccinated with a live attenuated tetravalent vaccine, T cell epitopes were mapped in several regions of the DENV proteome, although CD8^+^ T cells preferentially recognized NS3, NS5, and NS4b regions, while CD4^+^ T cells tended to recognize structural proteins and NS1^51–55^. Similarly, the same profile was detected in JEV infection^47^ and YFV vaccination^49,56^. In patients infected with ZIKV, CD4^+^ T cells target structural and nonstructural proteins in equal proportions, while CD8^+^ T cells preferentially focus on structural proteins^24,50^. In the context of previous exposure to DENV^54^, the CD8^+^ T cell response is modulated towards nonstructural proteins. A study with a DENV-naïve/ZIKV-infected patient, CD4^+^ and CD8^+^ T cell responses target preferentially NS2A and envelope proteins, respectively^57^. Furthermore, murine models have been instrumental not only in understanding the role of cellular immunity during flavivirus infections, but also in determining the epitopes recognized by T cells. Immunization of AG129 mice (Ifnar1−/−, Ifngr1−/−) and human HLA class II transgenic mice revealed that CD4^+^ T cell responses were directed to NS1, NS3, NS5 and envelope proteins^58^. A similar profile of CD8^+^ T cell responses was detected in infected C57BL/6 mice^24^. In this work, we identified four peptides present in the envelope region of the virus (ZIKV1(E_1–20_), ZIKV6(E_51–70_), ZIKV36(E_351–370_) and ZIKV37(E_361–380_)), capable of inducing a cellular immune response to the H-2K^d^ and H-2K^b^ haplotypes. Previous work^58^ mapped different ZIKV-immunodominant epitopes in HLA class II transgenic mice after immunization and the peptide ZIKV1(E_1–20_), the same we mapped (Supplementary Fig. 3a), was presented by HLA-DR1, -DR4, and DR1501 and -DQ8. Also, epitope mapping was performed during ZIKV infection in H-2^b^ mice. Five CD8 immunodominant peptides were mapped^24^: two within peptide ZIKV1 (E_1–20_) (IGVSNRDFV and SNRDFVEGM), two in ZIKV6 (E_51–70_) region (TTVSNMAEV and RSYCYEASI) and one in the ZIKV36 (E_351–370_) region (MAVDMQTLTPV). In addition, one CD4-immunodominant peptide^25^ was also mapped in the middle of ZIKV36 (E_351–370_) and ZIKV37 (E_361–380_) peptide regions (PVGRLITANPVITES) (Supplementary Fig. 3a).

Collectively, our results demonstrate that E_ZIKV_, EDI/II_ZIKV,_ and EDIII_ZIKV_ proteins are highly antigenic and immunogenic, inducing specific humoral and cellular immune responses. Furthermore, epitope mapping during immunization allowed the identification of immunodominant epitopes. In summary, our work provides a detailed assessment of the post-immunization immune response in different strains of mice mapping T-specific recognition regions in the envelope protein of ZIKV. These findings could help to better understand the immune response against ZIKV and add valuable information for future vaccine design.

## Materials and methods

### Production of optimized E_ZIKV_, EDI/II_ZIKV_ and EDIII_ZIKV_ sequences

The alignment of ZIKV envelope (E_ZIKV_) sequence (aa 291–690 of the ZIKV polyprotein) was generated using 69 Brazilian ZIKV sequences by the software ClustalW (GenBank accession numbers are available at Supplementary Table 1). The codon optimized gene was synthesized (GenScript, NJ) and cloned into the pET21a vector (pET21a-E_ZIKV_). Then, the EDI/II_ZIKV_ ectodomain (aa 291–600) was amplified by PCR (primers sense 5′-GGGCTAGCATTCGTTGCATCG-3′ and anti-sense 5′-CCCTCGAGCGCGGTGCACAGGCTGTA-3′; and EDIII_ZIKV_ (aa 601–690) (primers sense 5′-GGGCTAGCGCGTTCACCTTTACCAAAATT-3′ and antisense 5′-GGCTCGAGCCAGTGGTGGGT-3′) using Phusion High Fidelity DNA Polymerase (New England Biolabs) as recommended by the manufacturer. The PCR product was cloned into the pJET1.2/blunt vector (Thermo Fisher Scientific), digested with endonucleases *NheI* and *XhoI* (New England Biolabs). We purified the digested fragment using PureLink Quick Plasmid DNA kit (Invitrogen) and then cloned in the pET21a vector using T4 DNA ligase enzyme (New England Biolabs).

### Expression and purification of E_ZIKV_, EDI/II_ZIKV,_ and EDIII_ZIKV_ proteins

E_ZIKV_, EDI/II_ZIKV_ and EDIII_ZIKV_ recombinant proteins were expressed as monomers as previously described^59^. Briefly, transformed *E. coli* BL21 (DE3) RIL strain was cultured at 37 °C under agitation (200 rpm). After addition of 0.01 mM isopropyl β-d-1 thiogalactopyranoside (IPTG, Sigma) the bacterial pellet was suspended and lysed in a high-pressure system. The recombinant proteins were purified using a nickel affinity chromatography Ni-Sepharose histidine-tagged resin (GE Healthcare) as recommended by the manufacturer. Analysis of purified recombinant E_ZIKV_, EDI/II_ZIKV_ and EDIII_ZIKV_ proteins were performed by electrophoresis using 15% SDS-PAGE gel under reducing conditions.

### Western blot

Purified recombinant E_ZIKV_, EDI/II_ZIKV_ and EDIII_ZIKV_ proteins (500 ng) were submitted to 15% SDS-PAGE electrophoresis under reducing conditions and then transferred to a nitrocellulose membrane (Hybond-C extra nitrocellulose—GE Healthcare). Next, membranes were blocked with PBS containing Tween 20 (PBST) (0.05% v/v), non-fat milk (5% w/v) and BSA (2.5% w/v), overnight at 4 °C. After each step, the membranes were washed 3 times with PBST. Then, the membranes were incubated with anti-his 6 × tag (1:5000—Thermo Fisher Scientific) for two hours at room temperature (rt). Nitrocellulose membranes were then incubated with HRP-labeled goat anti-mouse IgG (1:5000; KPL) at rt for 1 h. We used a chemiluminescence kit (ECL kit, GE Healthcare) to develop the reaction as recommended by manufacturer’s instructions and analyzed by Alliance 4.7 software (Uvitec; Cambridge).

### Dot blot

Purified recombinant proteins E_ZIKV_, EDI/II_ZIKV_, EDIII_ZIKV_ and BSA (Bovine Serum Albumin) were added on nitrocellulose membrane (1 μγ) in total volume of 10 µL. After the membranes were completely dry, the following steps were performed as described above with just a minor modification. The primary antibody used was the anti-flavivirus 4G2 (1 μg/mL).

### Mice and immunization

Female BALB/c or C57Bl/6 mice (6- to 8-weeks-old) were bred at Centro de Desenvolvimento de Modelos Experimentais para Medicina e Biologia (CEDEME)—UNIFESP. All mice were housed at Division of Immunology—UNIFESP. The experiments were approved by the UNIFESP Institutional Animal Care and Use Committee (IACUC) (protocol number #2020100418), in accordance with the recommendations of the Federal Law 11.794 (2008) and the Guide for the Care and Use of Laboratory Animals of the Brazilian National Council of Animal Experimentation (CONCEA). Mice were immunized with two doses, fifteen days apart, with equimolar amounts of E_ZIKV_ (10 μg), EDI/II_ZIKV_ (7.78 μg) or EDIII_ZIKV_ (2.44 μg) in the presence of poly (I:C) adjuvant (50 μg; Invivogen) in a total volume of 100 μL at the base of the tail (subcutaneously). The mice were bled by submandibular vein after each dose and were euthanized fifteen days after the second dose.

### Measurement of ZIKV-specific antibodies

For ELISA, 96-well plates (high binding, Costar) were coated at rt overnight with 250 ng/well of E_ZIKV_, EDI/II_ZIKV_ or EDIII_ZIKV_ diluted in 50 μL/well of PBS 1x. After each step the plates were washed with PBS Tween 20 (PBST) (0.02% v/v). Then, the plates were blocked for 2 h at rt with 150 μL of PBST, BSA (1% w/v) and non-fat milk (5% w/v). Next, serum from mice immunized were serially diluted and 100μL were applied to each well for 2 h at rt. Plates were then incubated with horseradish peroxidase-labeled goat anti-mouse IgG (1:10,000; KPL) for 2 h at rt. The enzymatic reaction was developed with 1 mg/mL of o-phenylenediamine (OPD, Sigma) diluted in phosphate–citrate buffer (0.2 M Na_2_PO_4_ and 0.2 M C_6_H_8_0_7_), pH 5, containing 0.03% (v/v) hydrogen peroxide and was stopped with 4 N H_2_SO_4_. We used a ELISA reader (EnSpire Multimode Plate Reader; PerkinElmer) to read plates at 492 nm (OD_492_). The antibody titer was determined by the highest dilution of serum that presented an OD_492nm_ between 0.1 and 0.2.

### Plaque Reduction Neutralization Test (PRNT)

A ZIKV isolate from Brazil (ZIKV^BR^), described by Cugola et al.^60^, was amplified in Vero E6 cells (ATCC CRL-1586) in complete MEM medium (supplemented with 10% FBS and 1% penicillin/streptomycin (GIBCO)) for 96 h. For the neutralization assay, 1 × 10^5^ Vero CCL-81 cells (ATCC CCL-81) were plated in 24-well plates (Costar) in complete MEM medium and incubated overnight at 37 °C with 5% CO_2_. The following day, serum samples from immunized mice were previously inactivated for 30 min at 56 °C and incubated in the presence of 100 Plaque Forming Units (PFU) of ZIKV. Then, serum samples were serially diluted in 2% MEM medium (containing 2% FBS, 1% penicillin/streptomycin (GIBCO)) and then incubated with 100 PFU of ZIKV per well, for 1 h at 37 °C with 5% CO_2_. In addition, we added a dose test (DT)—which corresponds to 100 PFU; DT50 (50 PFU), mock (cell only), serum from non-immunized control mouse and from ZIKV-infected patient. Then, cells were incubated with a mixture containing the serum-virus for 3 h at 37 °C. Subsequently, the cells were overlayed with MEM medium with CMC (1.6% carboxymethylcellulose (CMC, Sigma) containing 10% FBS, 1% penicillin/streptomycin, 0.05% Amphotericin B (Fungizone, Gibco)) and incubated at 37 °C. After 4 days, the medium with CMC was completely removed and washed twice with PBS1X. Cells were then fixed with 4% paraformaldehyde (Sigma), stained with crystal violet (0.2%, Sigma) for half an hour and the excess dye was removed with distilled water.

### Immunofluorescence assay (IFA)

The immunofluorescence assay was performed as described previously^59^. Briefly, 1 × 10^4^ ZIKV-infected Vero cells were added to a multi-well glass slides in a MOI 0.1 at rt for 1 h. Cells were then fixed with acetone 80% solution (v/v) and incubated at − 20 °C for 30 min. After each step, wells were washed 3× with PBS 1X. Following incubation for 30 min with primary antibody (mouse immune sera, 1:500), goat anti-mouse IgG conjugated with FITC (1:750; Sigma) was added for 30 min. Immunofluorescence assay was performed using fluorescence microscopy (Olympus BX21) and the images were captured by CellSens software.

### Splenocyte isolation

After euthanasia (2 weeks after the last dose) the spleens were aseptically removed, and ammonium chloride potassium (ACK) was used to lyse red blood cells. Splenocytes were resuspended in RPMI medium supplemented with 10% of fetal bovine serum, 40 μg/mL of gentamicin, 1% v/v vitamin solution, 2 mM l-glutamine, 1% v/v non-essential aminoacids solution, 1 mM sodium pyruvate, 1% penicillin/streptomycin and 5 × 10^–5^ M of 2-mercaptoethanol (all from Gibco).

### Peptides

A peptide library (39 peptides) comprising the ZIKV envelope protein consensus sequence was synthesized (GenScript USA Inc) with purity more than 75% (20 amino acids overlapping 12-mer). Peptides were resuspended in DMSO (10 mg/mL) and stored at − 20 °C and organized into an optimized matrix (Supplementary Table 2) using *DeconvoluteThis!* Software as described previously^28^.

### ELISpot assay

IFNγ producing cells were assessed using IFNγ ELISpot Ready-SET-Go! Kit (eBiosciences) as recommended by the manufacturer instructions. Briefly, ELISpot plates (MAIPS 4510, Millipore) were coated with IFNγ-capture antibody. After washes and blocking, splenocytes (3 × 10^5^ cells) were added and incubated with pooled or individual peptides (10 μg/mL); equimolar amounts of recombinant proteins (E_ZIKV,_ EDI/II_ZIKV,_ EDIII_ZIKV_) or R10 (negative control). We used the AID ELISpot Reader System (Autoimmun Diagnostika GmbH, Germany) to count the number of spots. The number of IFNγ producing cells from stimulated wells were subtracted from the non-stimulated wells.

### T cell proliferation and cytokine production

To assess ZIKV-specific T cell proliferation, isolated splenocytes were labeled with carboxyfluorescein succinimidyl ester (CFSE), as previously described^59^. Briefly, splenocytes were labeled with 1.25 μM of CFSE (Molecular Probes), pouring the tube every two minutes for 10 min at 37 °C. Cells were then washed, resuspended and cultured for 5 days in the presence of the different stimuli. After 5 days, cells were first washed with buffer containing PBS with 0.5% BSA and 2 mM EDTA (FACS Buffer) and then stained with anti-mouse CD3-APCCy7 (clone 145-2C11), CD4-Pacific Blue (clone RM4-5) and CD8-APC (clone 53–6.7). For specific intracellular cytokine detection, splenocytes were cultured with the same antigens in the presence of anti-CD28 (2 μg/mL, BD Pharmigen) for second activation signal and Brefeldin A GolgiPlug™ (BD Pharmigen) for protein transport inhibition. Next, cells were washed with FACS buffer and stained with anti-mouse CD3-APCCy7 (clone 145-2C11), CD4-PerCP (clone RM4-5) and CD8-Pacific Blue (clone 53–6.7). Cells were then fixed and permeabilized using Cytofix/Cytoperm™ kit (BD Pharmigen*)* and washed with Perm/Wash buffer (BD Pharmigen). Cells were stained with anti-mouse TNFα-PECy7 (clone MP6-XT22) and IFNγ-APC (clone XMG1.2). All antibodies used for flow cytometry were from BD Pharmingen. The samples were acquired using the FACSCanto II flow cytometry (BD Biosciences) and analyzed using FlowJo software (Tree Star). To allow proper compensation, unstained and all single-color controls were performed. The frequency of proliferating cells was calculated by subtracting the values from unstimulated cells.

### Data analysis

Data normality tests were performed in GraphPad Prism including Shapiro–Wilk and D’Agostino-Pearson omnibus tests. Statistical significance (*p*-values) was calculated by One-way or Two-way ANOVA followed by Tukey honestly significantly different (HSD) post hoc test. Statistical analysis and graphical representation were performed using GraphPad Prism version 9.0 software.

### Ethics statement

This study was carried out in compliance with the recommendations of the Federal Law 11.794 (2008), the Guide for the Care and Use of Laboratory Animals of the Brazilian National Council of Animal Experimentation (CONCEA) and the ARRIVE guidelines (https://arriveguidelines.org). The protocol (number 2020100418) was approved by the UNIFESP Animal Care and Use Committee (IACUC).

## Supplementary Information

Supplementary Information.
