# Supplementary material for: ZIKV-envelope proteins induce specific humoral and cellular immunity in distinct mice strains
Source: Sci Rep. 2022 Sep 21;12:15733. doi: 10.1038/s41598-022-20183-x (PMC9492693; doi:10.1038/s41598-022-20183-x)
Supplement: Supplementary file 1 — Supplementary Information. [file 41598_2022_20183_MOESM1_ESM.pdf]

ZIKV-ENVELOPE PROTEINS INDUCE SPECIFIC HUMORAL AND CELLULAR IMMUNITY IN DISTINCT MICE STRAINS

Victória Alves Santos Lunardelli, Juliana de Souza Apostolico, Higo Fernando Santos Souza, Fernanda Caroline Coirada, Jéssica Amaral Martinho, Renato Mancini Astray, Silvia Beatriz Boscardin, Daniela Santoro Rosa.

Supplementary Figure Legends

Figure S1: Expression, purification, and characterization of recombinant E_{ZIKV}, EDI/II_{ZIKV} and $EDIII_{ZIKV}$. (a) Schematic diagram of recombinant ZIKV-envelope proteins. **(b)** SDS-PAGE analysis under reduction conditions of the recombinant proteins expressed as monomers. **(c)** Western blot analysis with anti-6xHis tag. (d) Dot blot analysis with anti-flavivirus 4G2 monoclonal antibody. MW, Molecular weight in kDa.

Figure S2: Specificity humoral response induced after immunization with different recombinant ZIKV envelope proteins. Comparison of response against **(a)** the same antigen used for immunization or **(b)** different domains induced after two doses with recombinant proteins E_{ZIKV}, EDI/II_{ZIKV} and $EDIII_{ZIKV}$ in the presence of poly (I:C) adjuvant in BALB/c and C57Bl/6 mice. Data represent mean $\pm$ SEM of **(a)** 4 or **(b)** 2 independent experiments. Statistical significance was measured by One-way ANOVA followed by Tukey's post hoc test, *$p<0.05$, **$p<0.01$, ***$p<0.001$, ****$p<0.0001$.

Figure S3. Mapped peptide sequences and flow cytometry analysis of proliferation and intracellular cytokine production. **(a)** Representation of four mapped peptides on ZIKV envelope protein sequence. Peptides mapped by other groups were also represented in the figure[24,25,58](figure made using Snapgene software, www.snapgene.com); **(b)** Representative dot plots of a seven-color flow cytometry panel used for the detection of $CD3^+CD4^+$ or $CD3^+CD8^+$

cells that **(i)** proliferate or **(ii)** produce IFNγ and/or TNFα after *in vitro* stimulation with ZIKV-envelope recombinant proteins or ZIKV-peptides. After gating on cytokine-producing cells, Boolean combinations were then created using FlowJo software to determine the frequency of each response based on all possible combinations of cytokine-producing T cells.

Figure S4. Immunization with recombinant ZIKV envelope proteins induces cytokine-producing CD4$^+$ and CD8$^+$ T cells. Analysis of cytokine-producing cells after immunization of **(a)** BALB/c or **(b)** C57Bl/6 mice with two doses, with an interval of fifteen days, with equimolar amounts of the recombinant proteins E_{ZIKV}, EDI/II_{ZIKV} and $EDIII_{ZIKV}$ in the presence of the poly (I:C). Fifteen after the second dose, the spleen of each animal was removed and cultured in the presence of equimolar amounts of recombinant proteins or 5μg/mL of the individual peptides. For the detection of cytokine-producing T cells, the cells were restimulated on the 4th day for 12 hours in the presence of recombinant proteins, anti-CD28 and brefeldin A. The cells were stained with anti-CD3, -CD4 and -CD8, then permeabilized and labeled for intracellular cytokines (representative gating strategies shown in Supplementary Figure 3b). The frequency of cells that produce cytokines was calculated by subtracting the values from the unstimulated cell culture. Statistical significance was measured by Two-way ANOVA followed by Tukey's post hoc test, *$p<0.05$, **$p<0.01$, ***$p<0.001$, ****$p<0.0001$. Data represent mean $\pm$ SEM of 2 independent experiments.

Supplementary Figure 1

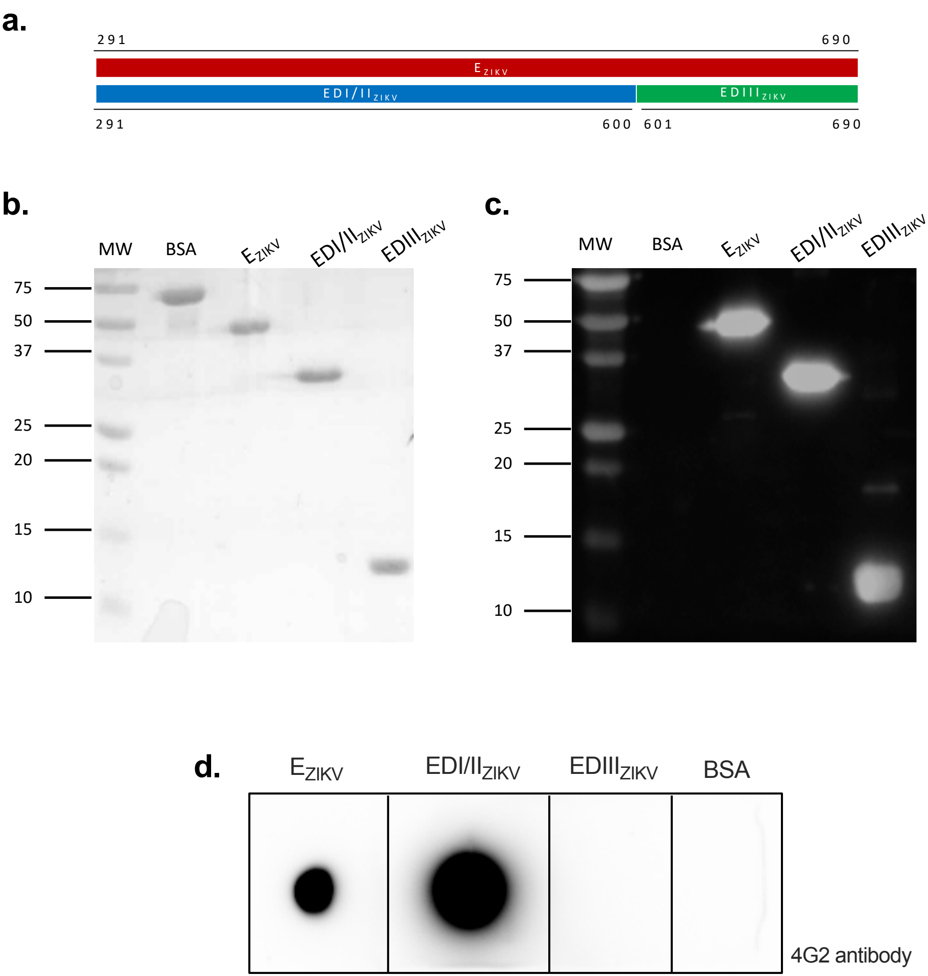

Supplementary Figure 2

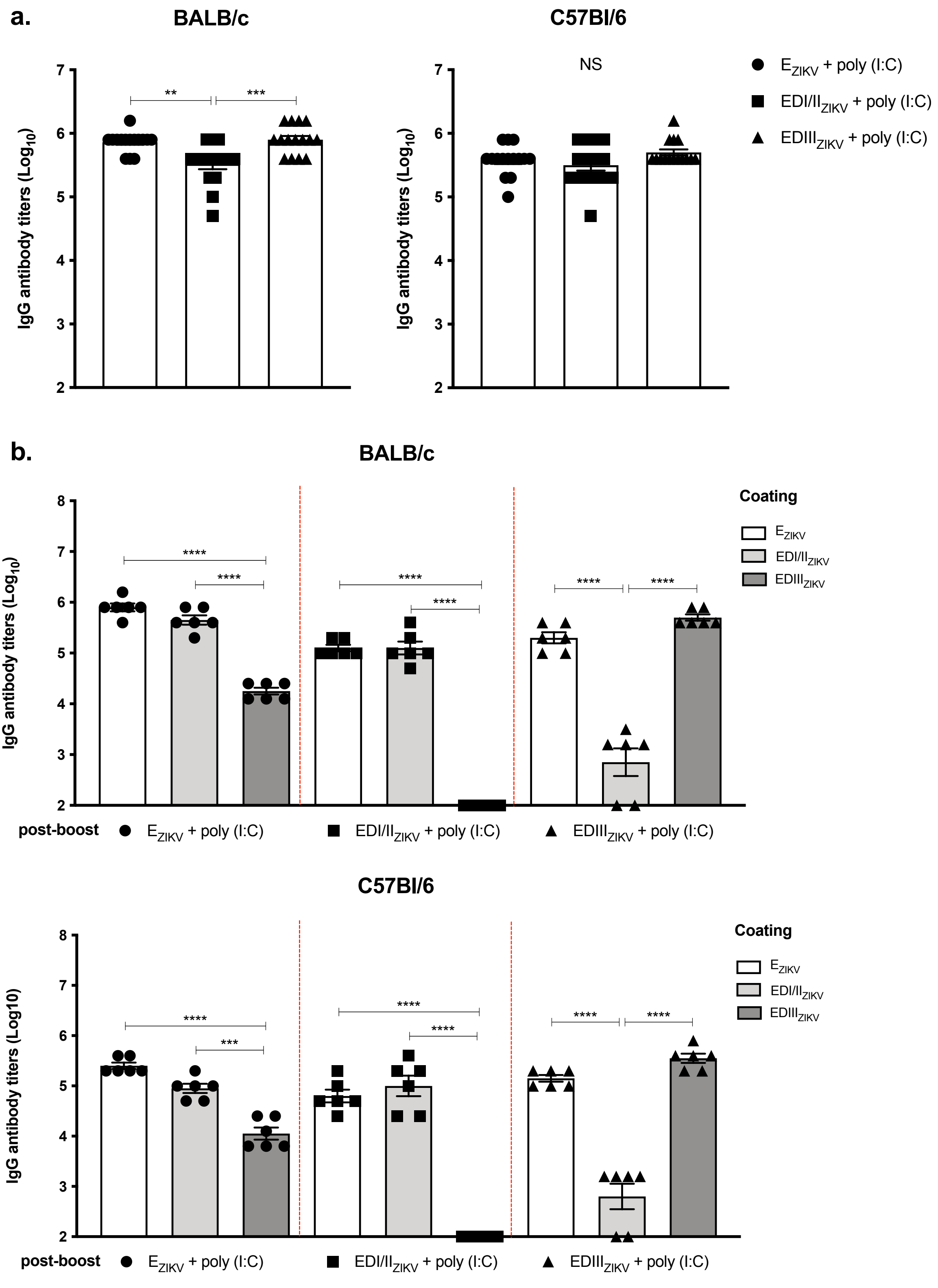

Supplementary Figure 3

a. ZIKV-epitope sequence

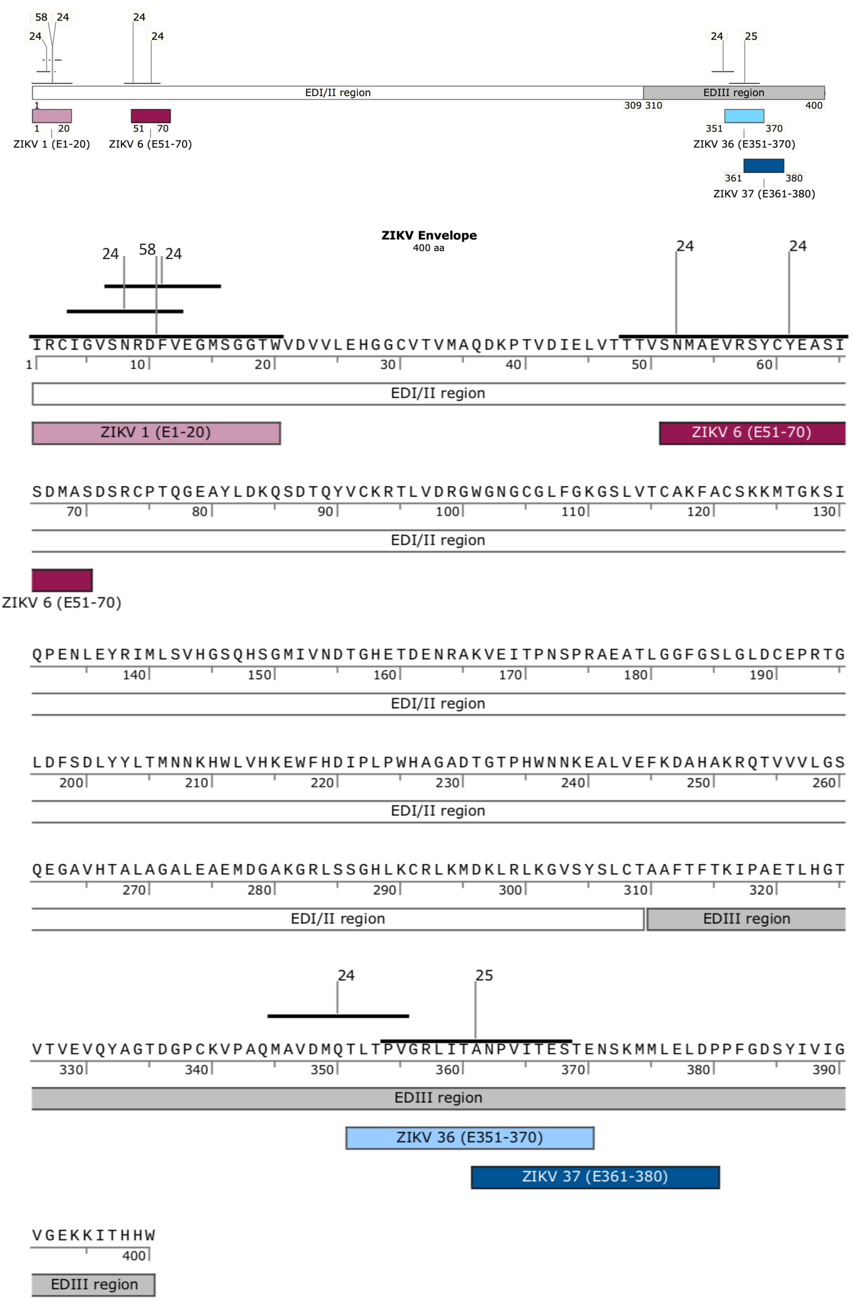

b. Representative Gating Strategies

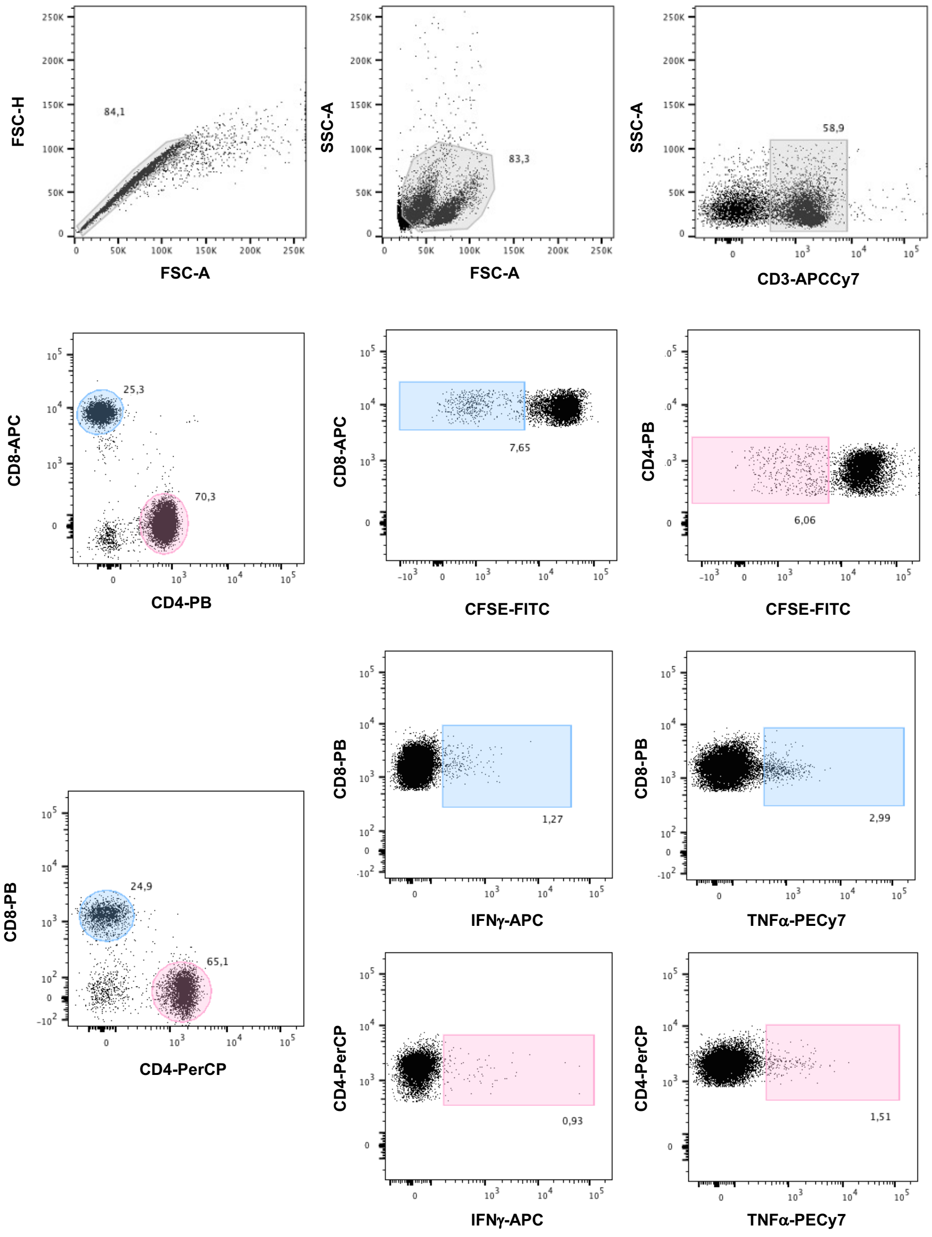

Supplementary Figure 4

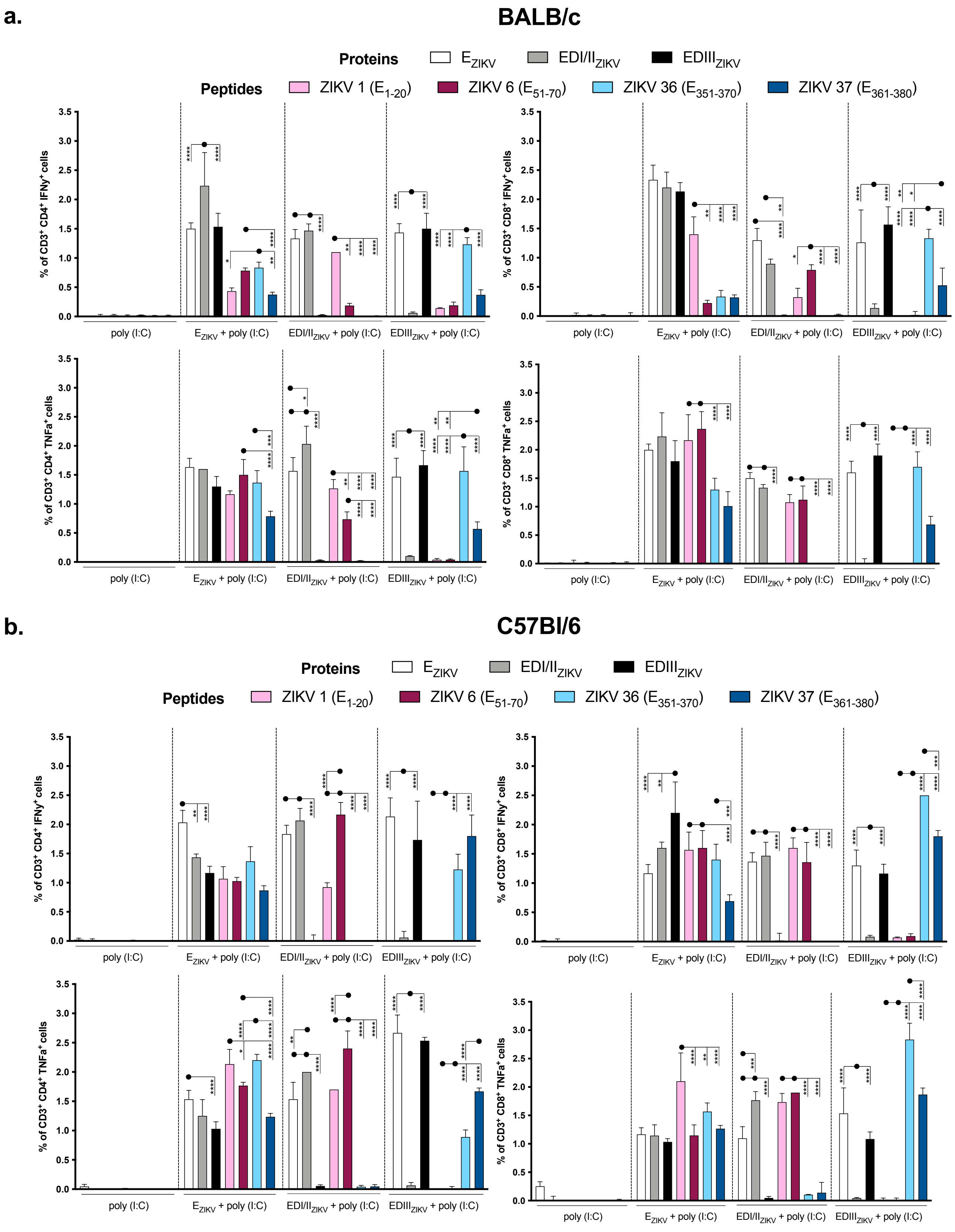

Supplementary Table 1. Brazilian ZIKV isolate sequences.

Zika virus isolate	*GenBank accession number*
BeH815744	KU365780.1
BeH818995	KU365777.1
BeH819015	KU365778.1
BeH819966	KU365779.1
SSABR1	KU707826.1
ZBRA105	KY558989.1
FC-DQ62D1-PLA	KY785480.1
FC-DQ60D1-URI	KY785479.1
FC-DQ62D2-URI	KY785455.1
FC-6863-SER	KY785450.1
FC-DQ5D1-URI	KY785437.1
FC-6706-SER	KY785433.1
FC-DQ58D1-PLA	KY785427.1
FC-DQ75D1-URI	KY785426.1
FC-6418-SER	KY785410.1
FC-DQ42D1-URI	KY014320.2
FC-6696-SER	KY014313.2
FC-DQ49D1-PLA	KY014307.2
FC-DQ107D1-URI	KY014301.2
FC-6864-URI	KY014297
FC-DQ131D1-URI	KY014296.2
BR_SJRP1840	KY441403.1
BR_SJRP61	KY441402.1
BR_SJRP21	KY441401.1
ZBRY6	KY559027.1
ZBRX128	KY559022.1
ZBRX127	KY559021
ZBRX100	KY559017.1
ZBRX16	KY559016.1
ZBRX13	KY559013.1
ZBRX12	KY559012.1
ZBRX11	KY559011.1
ZBRX8	KY559010.1
ZBRX6	KY559008.1
ZBRX4	KY559007.1
ZBRX1	KY559005.1
ZBRC319	KY558999.1
ZBRC313	KY558998.1
ZBRC301	KY558995.1
Rio-U1	KU926309.2
BR/AM/16800005	KY631492.1

Brazil_2015_MG	KX811222.1
15261	MF073358.1
16288	MF073357.1
9	KX197205.1
Paraiba_01	KX280026
RIO-BM1	KY272991.1
Bahia02	KX101060.1
Bahia07	KU940228.1
HS-2015-BA-01	KX520666.1
Natal RGN	KU527068.1
SPH2015	KU321639.1
BeH823339	KU729217.2
ZBRE69	KY559004.1
PE243	MF352141.1
ZBRC14	KY558990.1
17829	KR872956.1
PE243/2015	KX197192.1
ZBRC302	KY558996.1
15098	MF073359.1
Bahia09	KU940224.1
ZKV2015	KU497555.1
ZBRX15	KY559015.1
FC-DQ47D1-PLA	KY014309.1
BeH818305	KU729218.1
FC-DQ122D1-PLA	KY785456.1
Rio-S1	KU926310.2
FC-DQ28D1-URI	KY014317.2
ZIKV-GB-BR	KX830930.1

Supplementary Table 2. List of E_{ZIKV} protein derived peptides.

Peptide	*Position**	*Sequence***	*Domain*	*Pool*
ZIKV 1	1-20	IRCIGVSNRDFVEGMSGGTW	EDI + EDII	1, 6
ZIKV 2	11-30	FVEGMSGGTWVDVVLEHGGC	EDI + EDII	1, 6
ZIKV 3	21-40	VDVVLEHGGCVTVMAQDKPT	EDI + EDII	1, 7
ZIKV 4	31-50	VTVMAQDKPTVDIELVTTTV	EDI + EDII	1, 7
ZIKV 5	41-60	VDIELVTTTVSNMAEVRSYC	EDI + EDII	1, 8
ZIKV 6	51-70	SNMAEVRSYCYEASISDMAS	EDI + EDII	1, 8
ZIKV 7	61-80	YEASISDMASDSRCPTQGEA	EDI + EDII	1, 9
ZIKV 8	71-90	DSRCPTQGEAYLDKQSDTQY	EDI + EDII	1, 9
ZIKV 9	81-100	YLDKQSDTQYVCKRTLVDRG	EDI + EDII	2, 6
ZIKV 10	91-110	VCKRTLVDRGWGNGCGLFGK	EDI + EDII	2, 6
ZIKV 11	101-120	WGNGCGLFGKGSLVTCAKFA	EDI + EDII	2, 7
ZIKV 12	111-130	GSLVTCAKFACSKKMTGKS	EDI + EDII	2, 7
ZIKV 13	121-140	CSKKMTGKSIQPENLEYRIM	EDI + EDII	2, 8
ZIKV 14	131-150	QPENLEYRIMLSVHGSQHSG	EDI + EDII	2, 8
ZIKV 15	141-160	LSVHGSQHSGMIVNDTGHET	EDI + EDII	2, 9
ZIKV 16	151-170	MIVNDTGHETDENRAKVEIT	EDI + EDII	2, 9
ZIKV 17	161-180	DENRAKVEITPNSPRAEATL	EDI + EDII	3, 6
ZIKV 18	171-190	PNSPRAEATLGGFGSLGLDC	EDI + EDII	3, 6
ZIKV 19	181-200	GGFGSLGLDCEPRTGLDFSD	EDI + EDII	3, 7
ZIKV 20	291-210	EPRTGLDFSDLYYLTMNNKH	EDI + EDII	3, 8
ZIKV 21	201-220	LYYLTMNNKHWLVHKEWFHD	EDI + EDII	3, 9
ZIKV 22	211-230	WLVHKEWFHDIPLPWHAGAD	EDI + EDII	3, 10
ZIKV 23	221-240	IPLPWHAGADTGTPHWNNKE	EDI + EDII	3, 10
ZIKV 24	231-250	TGTPHWNNKEALVEFKDAHA	EDI + EDII	3, 10
ZIKV 25	241-260	ALVEFKDAHAKRQTVVVLGS	EDI + EDII	4, 6
ZIKV 26	251-270	KRQTVVVLGSQEGAVHTALA	EDI + EDII	4, 7
ZIKV 27	261-280	QEGAVHTALAGALEAEMDGA	EDI + EDII	4, 8
ZIKV 28	271-290	GALEAEMDGAKGRLSSGHLK	EDI + EDII	4, 9
ZIKV 29	281-300	KGRLSSGHLKCRLKMDKLRL	EDI + EDII	4, 10
ZIKV 30	291-310	CRLKMDKLRLKGVSYSLCTA	EDI + EDII	4, 10
ZIKV 31	301-320	KGVSYSLCT**AAFTFTKIPAE**	EDI + EDII + EDIII	4, 10
ZIKV 32	311-330	**AFTFTKIPAETLHGTVTVEV**	EDIII	4, 10
ZIKV 33	321-340	**TLHGTVTVEVQYAGTDGPCK**	EDIII	5, 6
ZIKV 34	331-350	**QYAGTDGPCKVPAQMAVDMQ**	EDIII	5, 7
ZIKV 35	341-360	**VPAQMAVDMQTLTPVGRLIT**	EDIII	5, 7

ZIKV 36	351-370	**TLTPVGRLITANPVITESTE**	EDIII	5, 8
ZIKV 37	361-380	**ANPVITESTENSKMMLELDP**	EDIII	5, 8
ZIKV 38	371-390	**NSKMMLELDPPFGDSYIVIG**	EDIII	5, 9
ZIKV 39	381-400	**PFGDSYIVIGVGEKKITHHW**	EDIII	5, 9

*Amino acid position on E_{ZIKV} protein
**Bold represents $EDIII_{ZIKV}$ amino acid sequence
